# Age-related dynamics of predominant methanogenic archaea in the human gut microbiome

**DOI:** 10.1186/s12866-025-03921-9

**Published:** 2025-04-04

**Authors:** Rokhsareh Mohammadzadeh, Alexander Mahnert, Tejus Shinde, Christina Kumpitsch, Viktoria Weinberger, Helena Schmidt, Christine Moissl-Eichinger

**Affiliations:** 1 Diagnostic and Research Institute of Hygiene, Microbiology and Environmental Medicine, Medical University of Graz, Neue Stiftingtalstraße 6, Graz, 8010 Austria; 2 Division of Molecular Biology and Biochemistry, Medical University of Graz, Graz, Austria; 3 BioTechMed, Graz, 8010 Austria

**Keywords:** Aging, Gut microbiota, methanogenic archaea, Butyrate-producing bacteria, Metagenome

## Abstract

**Background:**

The reciprocal relationship between aging and alterations in the gut microbiota is a subject of ongoing research. While the role of bacteria in the gut microbiome is well-documented, specific changes in the composition of methanogens during extreme aging and the impact of high methane production in general on health remain unclear. This study was designed to explore the association of predominant methanogenic archaea within the human gut and aging.

**Methods:**

Shotgun metagenomic data from the stool samples of young adults (*n* = 127, Age: 19–59 y), older adults (*n* = 86, Age: 60–99 y), and centenarians (*n* = 34, age: 100–109 years) were analyzed.

**Results:**

Our findings reveal a compelling link between age and the prevalence of high methanogen phenotype, while overall archaeal diversity diminishes. Surprisingly, the archaeal composition of methanogens in the microbiome of centenarians appears more akin to that of younger adults, showing an increase in *Methanobrevibacter smithii*, rather than *Candidatus* Methanobrevibacter intestini. Remarkably, *Ca.* M. intestini emerged as a central player in the stability of the archaea-bacteria network in adults, paving the way for *M. smithii* in older adults and centenarians. Notably, centenarians exhibit a highly complex and stable network of these two methanogens with other bacteria. The mutual exclusion between *Lachnospiraceae* and these methanogens throughout all age groups suggests that these archaeal communities may compensate for the age-related drop in *Lachnospiraceae* by co-occurring with *Oscillospiraceae*.

**Conclusions:**

This study underscores the dynamics of archaeal microbiome in human physiology and aging. It highlights age-related shifts in methanogen composition, emphasizing the significance of both *M. smithii* and *Ca.* M. intestini and their partnership with butyrate-producing bacteria for potential enhanced health.

**Supplementary Information:**

The online version contains supplementary material available at 10.1186/s12866-025-03921-9.

## Background

Aging is a complex process that affects the physiological, metabolic, and immune functions of humans often leading to chronic inflammation and metabolic issues [1]. It is uncertain whether the observed changes in the microbiota are a cause or consequence of aging. According to previous studies, elderly and centenarians tend to have distinct gut microbiome profiles as the latter are able to rearrange the microbiota that contribute to host health and physiology. This includes mitigating the depletion of *Ruminococcaceae*, *Lachnospiraceae*, and *Bacteroidaceae* through the promotion of potential health-enhancing subdominant species like *Akkermansia*, *Bifidobacterium*, and *Christensenellaceae* [2–5]. It is however challenging to determine whether these microbial differences contribute to extreme aging or result from a healthier lifestyle [6]. Animal studies suggest that age-related microbial imbalances can impact lifespan, with some animals benefiting from supplementation with a microbiome of younger ones [7] and others experiencing intestinal problems and premature mortality due to aging-associated microbiome changes [8]. Despite uncertainties regarding whether gut dysbiosis is a cause or consequence of aging and the subsequent inflammatory disorders, maintaining gut microbiota homeostasis is believed to be crucial for healthy aging and potentially supportive of human longevity [9, 10].

Since short chain fatty acids (SCFAs) produced by the microbiota are absorbed into the host bloodstream through the intestinal epithelium, it is plausible that microbiota-derived metabolites could have a substantial impact on human longevity [11]. The decline in metabolic health observed in old age may partly result from altered levels of intestinal SCFAs, and in particular, butyrate, leading to the disruption of gut barrier integrity, increased vulnerability to infections, and affecting conditions like insulin sensitivity and energy expenditure [12]. The study by Biagi et al., which is one of the few studies on the microbiome of centenarians residing in Western Europe, revealed the changes in gut microbial composition of these subjects, characterized by a decrease in core abundant taxa like *Bacteroides*, *Roseburia*, and *Faecalibacterium* species, along with an increase in rare taxa. Interestingly, they also observed a change in the population of butyrate-producing bacteria among centenarians. This suggests the possibility that, to achieve longevity, a complex and pervasive remodeling, which includes alterations of gut microbiota, should occur, favoring the balance between inflammatory and anti-inflammatory processes [13].

The human microbiome is not solely composed of bacteria; methanogenic archaea also play a significant but often overlooked role [14]. However, our understanding of age-related changes in gut-associated methanogens is limited. It is known that *Methanobrevibacter smithii*, as the predominant archaeal species within the human gut, gradually becomes the dominant archaeal colonizer in early life, with reported higher relative abundances in centenarians [13]. Moreover, Methanomassilicoccales have been frequently observed in elderlies as compared to younger adults (prevalence of 40% vs. 10%) [15], and similarly, a significant age-related upward trajectory was observed for *Methanomassiliicoccus luminyensis* and *Candidatus* Methanomassiliicoccus intestinalis [16]. Yet, the specific alterations in the composition of methanogens and their co-occurrence between bacterial members of the human gut during extreme aging remains unknown. It is noteworthy that a considerable proportion of the human population, approximately 20% of Western adults, falls into the category of high methane emitters, which have been characterized by a 1000-fold increase in *M. smithii* relative abundance in their gut microbiome. While an association between high methane emissions and complexity within the gastrointestinal microbiome of younger populations has been reported [17], the precise distribution of subjects with high methanogen phenotype and their microbiome across various age groups remains an aspect yet to be elucidated.

Furthermore, recently, the existence of two distinct species within *Methanobrevibacter smithii* has been suggested [18]. Indeed, a recent study of archaeal metagenome-assembled genomes (MAGs) underscores the pronounced dissimilarities between *M. smithii_A* and *M. smithii* genomes within the Genome Taxonomy Database (GTDB), to the extent that these distinctions fulfill the threshold criteria for species differentiation, as stipulated by the average nucleotide identity (ANI) metric (> 95%). Consequently, *M. smithii_A* has been designated as ‘*Candidatus* Methanobrevibacter intestini’ [18]. It remains an open question whether the distribution of *Ca.* M. intestini within the human population varies with age and whether there is a contribution of this archaeal species to methane production, analogous to its counterpart.

In the scope of this study, we sought to discern the diversity and distribution patterns of methanogenic archaea across different age groups of adults. Our study also encompasses a comprehensive examination of the prevalence for a high methanogen phenotype, within varying age groups. In addition, we embark on an exploration of the potential implications and associations of the presence of high methanogen phenotype in the context of extreme longevity. This research provides invaluable insights into the intricacies of archaeal dynamics within the human microbiome, and their age-related patterns.

## Materials and methods

### Study population

To include different age groups, our study incorporated three cohorts. As a young adult cohort, we used fecal samples from 91 subjects enrolled in Graz, Austria, which were initially collected for a study by Kumpitsch et al. (Cohort A) [17]. To encompass the older adults, a total of 94 participants aged 46–86 (68 ± 9.5) years (female: 51.6%) (Cohort B) were recruited at the Medical University of Graz, and finally, to include centenarians (Cohort C) in our study, we chose the metagenomes available in the Sequence Read Archive (SRA) repository under BioProject number PRJNA553191 from the study of Rampelli et al. [19], due to the close proximity of the subjects (Emilia Romagna region, Italy) to those enrolled for the first two cohorts.

### Sample collection and DNA extraction

Following collection, stool samples were promptly placed in a stool collection tube and immediately placed on ice. Subsequently, the samples were divided into separate Eppendorf tubes, suspended in a solution of approximately 0.1 gram of fecal material and 0.9% (w/v) DNA-free phosphate-buffered saline, and stored at -20 °C for subsequent analyses.

Genomic DNA extraction was carried out on 250 µl of fecal samples using the DNeasy PowerSoil Kit (QIAGEN, USA) according to the manufacturer’s protocol with a slight modification as previously described [17]. DNA was eluted in 80 µl elution buffer and the concentration was measured using the Qubit dsDNA HS Assay Kit (Thermo Fisher Scientific, USA).

### Metagenomic sequencing

Extracted DNA from fecal samples, was sent for sequencing to Macrogen (Seoul, South Korea). Libraries were generated via Nextera XT DNA Library construction kit and sequenced on NovaSeq 6000 Illumina platform with a read length of 151. Raw reads were obtained in fastq format with a mean read count of 27,623,362 for each sample.

### Taxonomic classification

Reads were quality checked and human reads were removed as previously described [19]. In summary, quality of all reads was assessed with fastqc (v0.11.8) [20], and subsequently filtered with trimmomatic (v0.38) [21] (a minimal length of 50 bp and a Phred quality score of 20 in a sliding window of 5 bp was applied). To filter out the human-mapped reads after quality filtering against the human chromosome GRCh38, bowtie2 (v2.3.5) and samtools (v1.9) were employed [22]. Subsequently, bedtools (v2.29.0) was used to extract the fastq files from the bam files [23]. We then used Kraken2 v.2.1.2 [24] to profile these final quality filtered reads with the Unified Human Gastrointestinal Genome (UHGG v.2.0.1) database of genomes, which consists of more than 289.000 archaeal and bacterial genomes. In order to increase the specificity and to compensate for the chance of returning the incorrect lowest common ancestor (LCA) of all genomes, a confidence threshold of 0.3 was chosen for Kraken2 v.2.1.2. To determine the relative abundance of bacterial and archaeal species, the Kraken2 output was subjected to analysis using Bracken v.2.7 [25]. with default settings. The report files were then merged to obtain an abundance table of microbial species which was used for further analysis.

### Removal of the batch effect

Differences in experimental designs and sequencing protocols have the potential to impact the distribution of microbiome data [26]. Due to the inclusion of diverse age demographics within three distinct cohorts A, B, and C, and our aim to combine these cohorts for the comprehensive exploration of the microbiome in various age groups, we opted to mitigate the influence of unwanted batch variations arising from distinct study designs and sequencing protocols employed. For this purpose, to generate a batch-corrected table of microbiome read counts for further analyses, we utilized the ConQuR tool [27]. This tool effectively addresses read distribution through quantile regression and handles the presence or absence of microbes using conditional quantile regression. We used Cohort B as the reference batch since it removed batch effects the most (the least PERMANOVA R2) and used it across all taxa to keep the overall composition of microbiome and used the presence of high methanogen phenotype (defined based on *Methanobrevibacter* relative abundance as described previously [17]), age classification, and sex as variables.

### Statistical analyses and visualization

Relative and differential abundance of archaea and bacteria were plotted in R (R-Core-Team, 2022) using the ggplot2 package (v3.3.3). Differentially abundant taxa were defined by q2-ALDex2 [28, 29] in QIIME2 [30]. To display those taxa in boxplots in R (packages: ggplot2 [31], dplyr [32], reshape [33]) the data of relative abundance were first CLR (centered log-ratio) transformed in R [34]. For statistical analyses, IBM SPSS Amos v26 was used. The normal distribution of parameters was checked using the Shapiro-Wilk test for the selection of the suitable statistical test. Throughout the manuscript, uncorrected significance values are reported as *p*-values and Benjamini-Hochberg corrected *p*-values are termed as *q*-values.

### Co-occurrence analysis

The sparse nature of metagenomic data, which can be attributed to various factors, including sample variations and sequencing depth, presents a challenge when inferring co-occurrence patterns. These variables can introduce challenges in statistical analysis, potentially leading to false-positive results and misleading correlations. To address this issue, we applied a prevalence filter that excluded microbial species present in less than 20% of samples in each age group. We chose this prevalence threshold based on the number of reads for *Ca*. M. intestini to avoid losing this particular species of interest. This approach alleviated the impact of matrix sparsity on our results.

To infer species-level associations of *M. smithii* and *Ca.* M. intestini with other bacteria within the abundance matrix of each age group separately, we employed SparCC [35] within the SCNIC tool (Sparse Co-occurrence Network Investigation for Compositional data) [36]. This method for inferring microbial associations incorporates the compositionality of microbiome data and considers the possibility of indirect correlations. Co-occurrence events with correlation of >|0.4| were visualized in Cytoscape v.3.10.0 where nodes represent taxa and edges represent positive and negative co-occurrences according to the SparCC R values. Betweenness and closeness centrality of nodes within the networks were also analyzed in Cytoscape.

### Gene catalog construction and analysis

Protein-coding genes were initially identified using Prodigal v.2.6.3 through the ATLAS workflow [37]. Elimination of duplicate genes was achieved through linclust with minid = 0.9 and coverag = 0.9 parameters [38]. The quantification of gene abundance per sample was performed using the combine_gene_coverages function within the ATLAS workflow, aligning high-quality filtered reads to the gene catalog via the BBmap suite v.39.01-1 [39]. Taxonomic and functional annotations were assigned based on the EggNOG database 5.0, employing eggnog-mapper (v.2.0.1) [40]. Subsequently, KEGG annotations were extracted from the output [41–43]. Read counts were implemented from the quality control workflow in ATLAS. The resultant outputs from ATLAS were analyzed in RStudio, following the procedures outlined in (https://github.com/metagenomce-atlas/Tutorial/blobs/master/R/Analyze_genecatalog.Rmd).

To ensure comparability of mapped read fractions for each sample, genes with annotations were acquired and normalized using the median of ratios method through DESeq2 [44]. Prefiltering was applied to retain only rows with a count of at least 10 for a minimum number of samples, which was determined based on the number of subjects with high methanogen phenotype. Furthermore, a differential expression analysis was conducted between subjects exhibiting a high methanogen phenotype and other subjects, employing the DESeq2 package in R Studio.

### Gene correlation with taxonomic information

The normalized abundance of genes was then correlated with the CLR transformed abundance of species from the shotgun sequencing data in R. This analysis was performed separately for each age group after the application of ConQuR on the count data. Only the genes of interest coding for enzymes responsible for butyrate and propionate formation were correlated with the taxonomic data using Spearman rank correlations [45]. The analysis was plotted in a heatmap using ggplot2.

## Results

### Study overview

We studied three distinct cohorts; cohort A, consisting of young adults recruited in Graz, Austria (*n* = 91, ages 19–37 years), cohort B consisting mostly of older adults also recruited in Graz, Austria (*n* = 94, ages 46–86 years) and cohort C with subjects enrolled in the Emilia Romagna (Italy) by Rampelli et al. [19] (*n* = 62, ages 22–109 years) which was geographically very close to the other cohorts and mostly included centenarians (Supplementary Fig. 1).

Overall, the study cohorts included a total of 247 subjects, of three age groups; 127 subjects aged 19–59 y (young adults, “YAs”), 86 subjects aged 60–99 y (older adults, “OAs”), and 34 subjects aged 100–109 (centenarians, “CENT”).

The analysis of age distribution among the three study groups (Cohort A, B, and C) revealed statistically significant differences, as expected. One-way analysis of variance (ANOVA) was conducted to compare the mean ages of participants in each cohort. The results indicated a significant variation in age across the cohorts (F = 391.323, *p* < 0.001), confirming the representation of different age groups by each cohort. On the other hand, a similar distribution of males and females existed within the investigated cohorts as shown by the chi-square test of independence (χ^2^ = 5.871, df = 2, *p* = 0.053).

It is important to note that the three cohorts under study may exhibit variations in numerous potential covariates that have remained unrecorded. Moreover, as differences in sample processing could result in bias in data analysis, we implemented a batch effect correction procedure based on the available metadata (see below).

### Removal of batch effect allows for comparison across datasets

The abundance profiling of the combined datasets based on UHGG showed a total of 4,253 distinct species, which were identified across 247 different samples. In order to remove the batch effects between studies and eradicate the high data variability, we employed the ConQuR. tool. Additionally, since all covariates (e.g., dietary information) could not be obtained for each subject in the study of Rampelli et al. [19], ConQur could to some extent account for these cofounders.

It was evident that ConQuR significantly diminished the study-related variation observed in the raw count data, as indicated by the Bray-Curtis and Aitchison distance analyses (Supplementary Fig. 2). PERMANOVA test showed no differences in the Bray-curtis and Aitchison distances before (Bray-curtis *p* = 0.001, Aitchison *p* = 0.001) and after (Bray-curtis *p* = 0.001, Aitchison *p* = 0.001) applying ConQur. However, when looking at the raw count scale, ConQur made significant adjustments to both the average values (centroids) and the spread of the data (size of the ellipses). Specifically, this tool aligned the averages of the three datasets to the same point, as depicted by the ellipses connecting the 95th percentile of points for each set in the bivariate plot (Supplementary Fig. 2).

Moreover, based on the PERMANOVA analysis on the count data, the initial R^2^ = 0.10026030 (before applying ConQuR), reduced to R^2^ = 0.01868353 (after applying ConQuR), which was lower than the effect of age (R^2^ = 0.03042544, after ConQuR vs. R^2^ = 0.05358378, before ConQuR) and effect of the presence of high methanogen phenotype (R^2^ = 0.02614169; after ConQuR vs. R^2^ = 0.03092168; before ConQuR).

### Aging is mirrored in the overall microbiome profile

After applying ConQuR for the 247 samples analyzed, the average number of raw reads for metagenomic analyses per subject from each age group ranged from 7,741,637, 6,993,479.8, and 10,103,384 for YAs, OAs, and CENT, respectively, with archaeal reads constituting 0.4972%, 0.4001%, and 0.9476% of all reads corresponding to each age classification in the mentioned order, indicating almost the highest distribution of archaeal read counts in CENT (Mann-Whitney U-test; YAs: OAs *p* = 0.07, YAs: CENT *p* = 0.219, OAs: CENT *p* = 0.463) (Supplementary Fig. 3A).

**Fig. 1:**
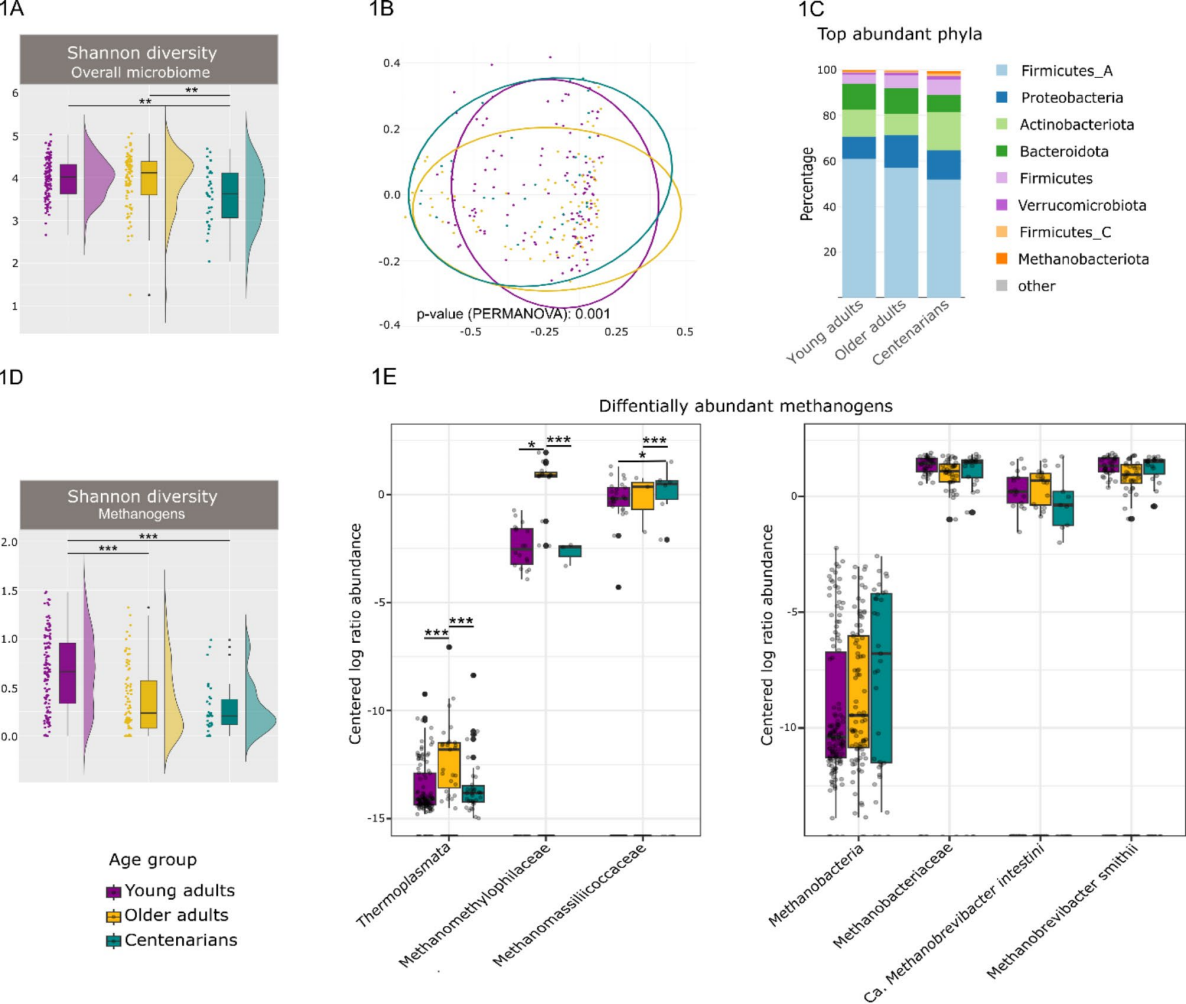
Comparative analysis of fecal microbiome diversity and methanogens in different age groups. (**A**) Comparison of Shannon diversity between three age groups of YAs, OAs, and CENT. (**B**) Beta diversity of fecal microbiomes between YAs, OAs, and CENT. (**C**) Stacked bar plot of relative abundances of the top microbial phyla is displayed by age groups (**D**) Shannon diversity of methanogens in different age groups of YAs, OAs, and CENT. (**E**) Box plot of CLR-transformed abundances of the methanogenic archaea in each age group (Methanobacteria: YAs: OAs *q* = 0.162, YAs: CENT *q* = 0.489; OAs: CENT *q* > 0.5; *Methanobacteriaceae*: YAs: OAs, *q* = 0.062; YAs: CENT *q* > 0.5; OAs: CENT, *q* > 0.5; *Methanobrevibacter smithii*: YAs: OAs *q* > 0.5, YAs: CENT *q* > 0.5; OAs: CENT; *Candidatus* Methanobrevibacter intestini: YAs: OAs *q* = 0.263; YAs: CENT *q* = 0.0905, OAs: CENT *q* > 0.5; Thermoplasmata: YAs: OAs, *q* < 0.001, YAs: CENT *q* = 0.00102 OAs: CENT *q* = 0.00102; *Methanomethylophylaceae*: YAs: OAs *q* < 0.001, YAs: CENT *q* > 0.5; OAs: CENT *q* < 0.001; *Methanomassiliicocaceae*: YAs: OAs q > 0.5; YAs: CENT q = 0.019; OAs: CENT q = 0.0013). Line in boxes is a median of index scores, boxes represent interquartile range, whiskers represent lowest and highest values, and dots represent each sample. Statistical significance in 1 A and 1D is indicated by **p < 0.001**p < 0.01 and *p < 0.05. Statistical significance levels of ALDEx2 test in 2E after adjustment for multiple comparison are indicated with ***q < 0.001, **q < 0.01, *q < 0.05

For alpha diversity analysis of the overall microbiome, we calculated the Shannon, richness, and evenness indices. The Shannon index of CENT was significantly lower than that of YAs (Wilcoxon test, *p* = 0.002) and OAs (Wilcoxon test, *p* = 0.005) (Fig. 1A). The same trend was observed for the evenness index as it was significantly lower in CENT compared to YAs (Wilcoxon test, *p* = 0.003) and OAs (Wilcoxon test, *p* = 0.002) (Supplementary Fig. 3B). The richness index decreased with age (Supplementary Fig. 3B); however, this was not significant (Supplementary Fig. 3B). These results suggest a lack of statistical divergence in the alpha diversity of the microbiota community between YAs and OAs; however, alpha diversity of CENT was significantly lower as compared with the other two age groups. This significant drop in the Shannon diversity measure of CENT was in line with observations made in previous studies [12, 46].

To characterize gut microbial patterns associated with aging, we also performed a β-diversity analysis using principal coordinates analysis (PCoA) and Non-Metric Multidimensional Scaling (NMDS). A major overlap was observed in the PCoA and NMDS plots, however, PERMANOVA under 999 permutations showed a significant difference (*p* < 0.001; stress (NMDS): 0.2577) between the beta-diversity of the three age groups (Fig. 1B, Supplementary Fig. 3C), as observed previously [6].

With respect to the bacteriome, we could observe that at phylum level, Firmicutes, Proteobacteria, Actinobacteriota, and Bacteroidota were the dominant bacterial taxa in each age group, which was in accordance with previous reports with different cohorts (Fig. 1C) [47]. However, both Firmicute_A and Bacteroidota exhibited significantly lower levels in CENT when contrasted with YAs and OAs (Firmicute_A *p* < 0.01, *q* < 0.01; Bacteroidota *p* < 0.001, *q* < 0.001; Wilcoxon test).

### The composition of methanogens in centenarians is similar to that of young adults

The potential impact of archaea, as the understudied members of the human microbiome, on aging was investigated in more detail. Aging affected the alpha diversity of methanogenic archaea as exhibited by the statistically significant decrease in the Shannon index (*p* < 0.001) across the studied age groups (Fig. 1D). This results is in contrast with previous reports based on 16S rRNA gene amplicon sequencing [15].

We observed distinct age-related shifts in the archaeome profile. Focusing first on the class Methanobacteria, there was a non-significant decline trend of increasing relative abundance with age [16, 48]. Within this class, the family *Methanobacteriaceae* showed a slight, non-significant decline in relative abundance in OAs compared to YAs and CENT (Fig. 1E, Supplementary Material 1). At the species level (same family), *Methanobrevibacter* spp. exhibited notable age-related differences: *Methanobrevibacter smithii* was less abundant in OAs compared to both YAs and CENT, while *Candidatus* Methanobrevibacter intestini, a newly identified species [18], increased in OAs relative to YAs before declining in CENT (Fig. 1E, Supplementary Material 1).

Shifting focus to the class Thermoplasmata, a statistically significant increase in relative abundance was observed in OAs compared to both YAs and CENT (Fig. 1E, Supplementary Material 1). This increase was primarily driven by two families: *Methanomethylophylaceae* and *Methanomassiliicocaceae*. *Methanomethylophylaceae* were significantly more abundant in OAs compared to YAs and CENT, while *Methanomassiliicocaceae* showed a more complex pattern. Although OAs had higher levels of *Methanomassiliicocaceae* than YAs, the difference was not significant. However, CENT exhibited significantly elevated levels compared to both YAs and OAs (Fig. 1E, Supplementary Material 1), consistent with previous studies.

In summary, while both OAs and CENT showed decreased methanogen diversity, the methanogenic archaea composition in CENT more closely mirrors that of YAs than OAs. This similarity is most evident at the family and species levels, particularly in the relative abundance of *Methanomethylophylaceae* and *Methanobacteriaceae* (at family level), and *M. smithii* (at species level), where patterns in CENT align more closely with those of YAs.

### The two predominant *Methanobrevibacter* species co-exist and demonstrate co-occurrence with health-associated bacterial species

**Fig. 2:**
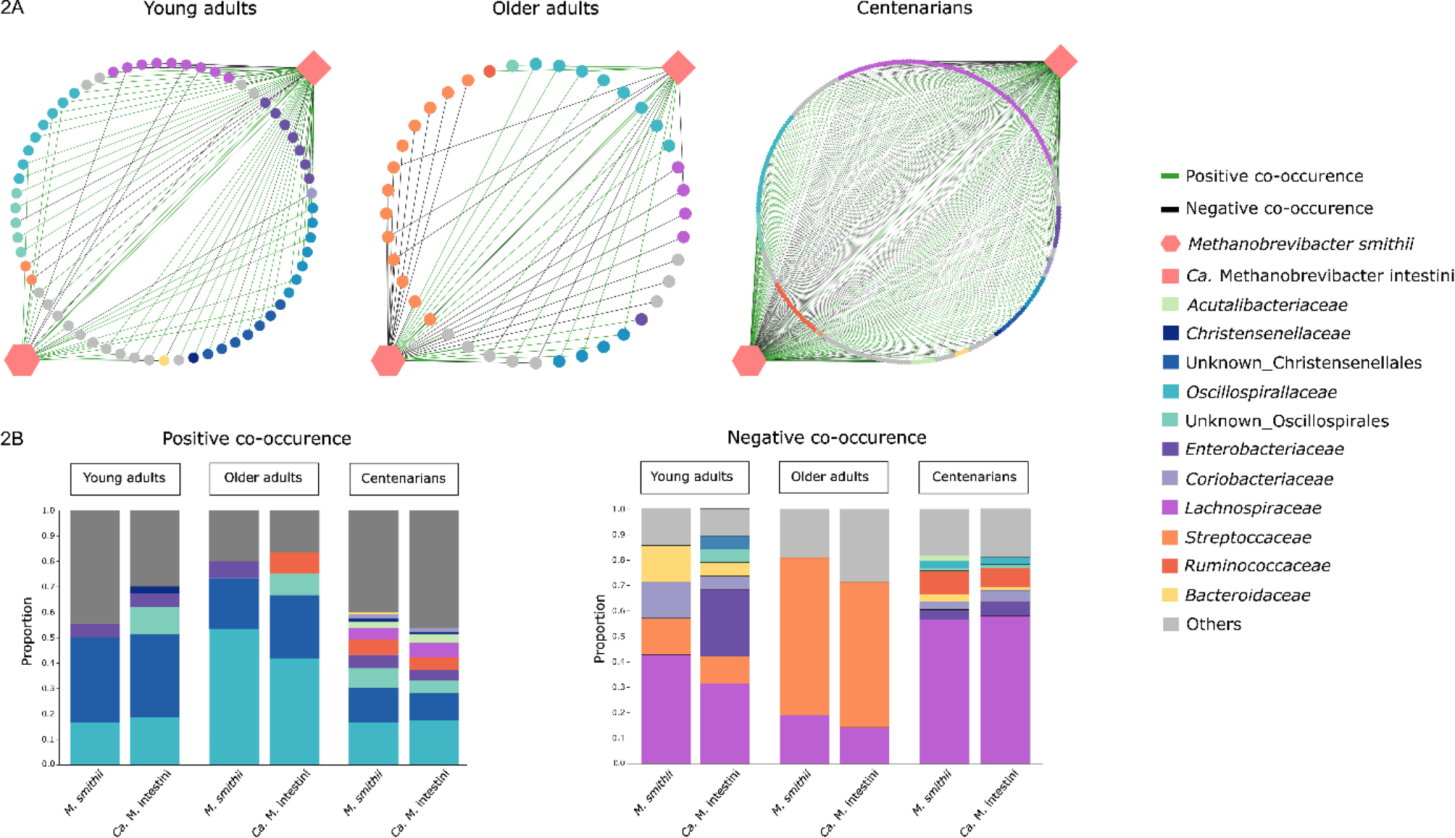
SparCC co-occurrence networks of *M. smithii* and *Ca.* M. intestini in all samples irrespective of the presence of high methanogen phenotype (**A**). Each node represents a single microbial species, and each edge a single association between a pair of microbial species. Positive and negative SparCC co-occurrences are indicated in green and black, respectively. These co-occurrences are elaborated in (**B**)

Microbial networks, which are constructed based on correlations in species abundances, offer insights into co-occurrences among microbes within a community. In order to gain a better understanding of the microbes with potential co-occurrence with *M. smithii* and *Ca.* M. intestini in the gut across different age groups, we utilized abundance data of taxa from each group to generate three distinct networks (Fig. 2A). Of note, while these networks reveal niche-sharing patterns, they do not necessarily indicate direct physical or biochemical interactions between microbes [49].

Our analysis of the networks revealed, that *M. smithii* and *Ca.* M. intestini had the highest degree of interconnectivity in CENT (Table 1), suggesting that these methanogens play a more central role in the gut microbiome of this age group. In contrast, OAs exhibited the lowest interconnectivity, pointing to a less complex network structure for these methanogens.

Centrality metrics, including betweenness and closeness centrality, as well as node degree, revealed a shift in the key drivers of these networks across age groups (Table 1). In YAs, *Ca.* M. intestini emerged as the keystone species, driving microbial networks, whereas in OAs and CENT, *M. smithii* took on this role (Table 1).

A detailed examination of microbial species in these networks highlights the consistent co-occurrence of *M. smithii* and *Ca.* M. intestini across all three age groups (Fig. 2A), which is unexpected given their similar resource requirements. The persistence of both species suggests that competition does not entirely exclude one from the niche, indicating a more complex dynamic between these two species.

Additionally, certain microbial taxa showed consistent associations with these methanogens. Members of the orders Oscillospirales and Christensenellales (specifically families *Oscillospiraceae* and *Christensenellaceae*) frequently co-occurred with both *M. smithii* and *Ca.* M. intestini in all age groups (Fig. 2A and B). This aligns with previous findings of positive associations between *Oscillospira* spp. and *Christensenellaceae* with *M. smithii* [17, 50].

However, the family *Lachnospiraceae* exhibited mixed associations. While species within this family, like *Roseburia hominis*, *Blautia hansenii*, and *Blautia massiliensis* positively co-occurred with *M. smithii* and *Ca.* M. intestini in CENT, a larger proportion of *Lachnospiraceae* members were associated with negative co-occurrences (mutual exclusion) across all age groups (Fig. 2B).

Age-specific patterns were also observed for other abundant bacterial taxa. For instance, *Streptococcus* emerged as a key taxon showing mutual exclusion with both *M. smithii* and *Ca.* M. intestini predominantly in OAs (Fig. 2B). This may reflect a shift toward “oralization” of the gut microbiome, possibly influenced by the use of proton pump inhibitors [51].

Furthermore, while some opportunistic pathogens within the family *Enterobacteriaceae* exhibited positive co-occurrences with *M. smithii* and *Ca.* M. intestini, mutual exclusion with pathogens like *Klebsiella*, *Salmonella*, and *Proteus* was mostly observed in YAs and CENT, suggesting a more protective role of these methanogens against harmful bacteria in YAs and CENT.

**Table 1:** Parameters of *M. smithii* and *Ca.* M. intestini networks with the gut microbial community of the studied subjects

Archaeal species	Network parameter	Network inference for all studied subjects			Network inference for subjects with high methanogen phenotype		
		YAs	OAs	CENT	YAs	OAs	CENT
*M. smithii*	Node degree (+/-) *	25 (18+, 7-)	37 (15+, 21-)	231 (132+, 98-)	82 (46 - /36 +)	117 (62+/55-)	169 (47-, 122+)
	Betweenness centrality	0.246	0.832	0.659	0.507	0.902	0.790
	Closeness centrality	0.627	0.911	0.871	0.442	0.678	0.734
*Ca.* M. intestini	Node degree (+/-) *	56 (37+, 19-)	19 (12+, 7-)	190 (121+, 69-)	156 (90 -/66+)	48 (27+/21-)	120 (60 +/60 -)
	Betweenness centrality	0.876	0.251	0.424	0.864	0.416	0.593
	Closeness centrality	0.901	0.661	0.770	0.626	0.421	0.649

* Positive (co-presence) /negative (mutually excluded) association

### High methanogen phenotype is twice as common in centenarians

Since the focus of our study was on the human archaeome and its association with aging, a phenotypic grouping of individuals according to their archaeal profile was necessary. A bimodal pattern has been demonstrated for the prevalence of *Methanobrevibacter* in the human gut, indicating that it is either highly prevalent or almost absent [52]. However, some subjects might still show a certain relative abundance of *Methanobrevibacter*, while not being categorized as high methanogen phenotype. To investigate how methanogens are linked with the overall microbiome composition, subjects were stratified into high and low methanogen phenotype based on the relative abundance of *Methanobrevibacter* according to our previous observations [17]. Interestingly, the prevalence of the subjects with high methanogen phenotype within distinct age groups was as follows: 25.2% (32/127; 95% CI: 19.6–31.6%) among YAs, 41.86% (36/86; 95% CI: 32.6–51.1%) among OAs, and 58.82% (20/34; 95% CI: 41.3–73.1%) among CENT (Supplementary Fig. 4A). This observation suggests an elevated prevalence of subjects with high methanogen phenotype with respect to aging. According to the results of Pearson’s chi-square test the prevalence percentages of subjects with high methanogen phenotype appear to be significantly different among different age groups (chi-square = 13.762, df = 2, *p* = 0.001027), and the presence of high methanogen phenotype and age group variables are significantly associated (chi-square = 15.458, df = 2, *p* < 0.001) (Supplementary Fig. 4B). In fact, based on these results, it was evident that the highest association between the presence or the frequency of high methanogen phenotype was in centenarians, while the reduced frequency of high methanogen phenotype was positively associated with YAs.

### The presence of high methanogen phenotype affects microbiome characteristics across age groups

**Fig. 3:**
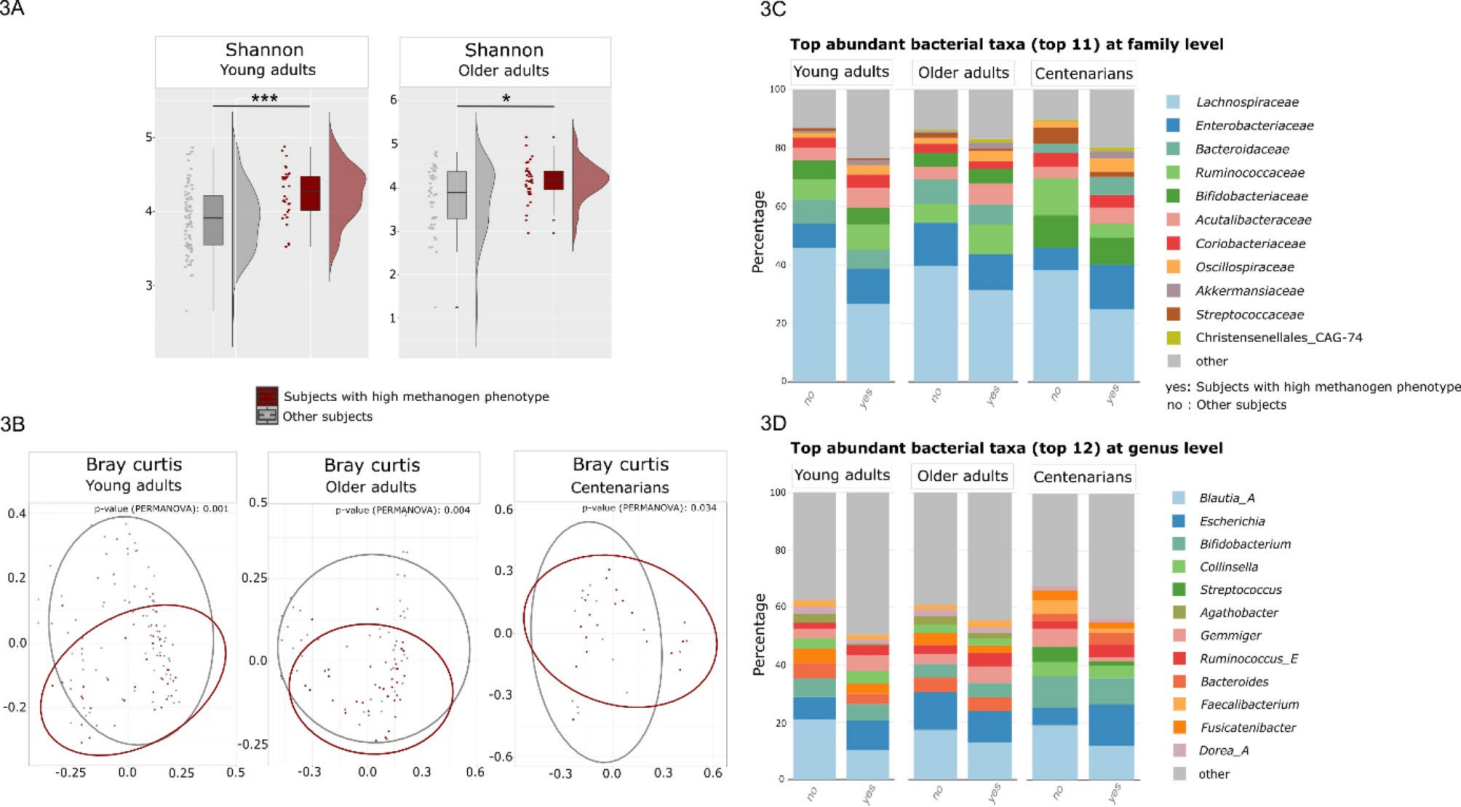
Differences in alpha and beta diversity, and top abundant taxa based on the metagenomics shotgun sequencing between subjects with high methanogen phenotype and other subjects in different age groups. (**A**) An examination of Shannon diversity index revealed significant differences in alpha diversity based on high methanogen phenotype in both YAs and OAs (***p < 0.001, *p < 0.05). However, all subjects either with or without high methanogen phenotype within CENT showed similar trends regarding Shannon index. (**B**) The microbiome of subjects with the high methanogen phenotype clustered significantly differently in the PCoA plots, regardless of age classification. However, this significance was notably higher only in YAs and OAs. (**C**) Bar chart of the most abundant bacterial taxa at family level and (**D**) genus level compared regarding the presence of high methanogen phenotype with respect to each age classification of YAs and OAs, as well as CENT

A significantly higher Shannon diversity was observed in subjects with high methanogen phenotype compared to those without in YAs and OAs (Fig. 3A) (Shannon index = 4.247 ± 0.353, *p* < 0.001 for YAs; Shannon index = 4.171 ± 0.265, *p* = 0.025 for OAs) (consistent with previous results [17]). There was also a consistent and statistically significant elevation in microbial richness attributed to the high methanogen phenotype, across all three age groups (*p* < 0.001; t-test), indicating a higher diversity of microbial signatures in the presence of high methanogen phenotype. The evenness measure, however, exhibited significant elevation with respect to the high methanogen phenotype only among YAs (*p* = 0.002; t-test) (Supplementary Fig. 5A).

The microbial community composition revealed statistically significant differentiation between clusters of subjects characterized by the presence of the high methanogen phenotype and those lacking it (within the age groups of YAs *p* = 0.001, OAs *p* = 0.004, and CENT *p* = 0.034) (Fig. 3B).

It is important to note that the variability within the microbial communities of subjects with high methanogen phenotype was notably higher in both YAs and OAs compared to CENT (YAs *p* < 0.001, OAs *p* = 0.005, CENT *p* = 0.028; PERMANOVA). The finding that subjects with high methanogen phenotype in CENT do not exhibit a strongly significant difference in their microbiome compared to those subjects within YAs and OAs might suggest that the gut microbiota of these subjects within the CENT might possess less unique microbiota as compared with those without high methanogen phenotype in this age group.

The results centered around the presence of the high methanogen phenotype revealed consistent variations in the relative abundance of some specific taxa, regardless of their categorization within a specific age group. Specifically, the family *Lachnospiraceae* and within this family, at genus level, *Agathobacter*,* Blautia*,* Dorea* (known butyrate-producing taxa) [53–55] were reduced in relative abundance in subjects with high methanogen phenotype across all age groups (Fig. 3B and C, Supplementary Fig. 5A, B, Supplementary Material 1). Additionally, diminished relative abundance of the family *Streptococcaceae* and the genus *Streptococcus* was documented in these subjects across all age groups (Fig. 3C, Supplementary Fig. 6A, B), while *Acutalibacteriaceae*, Christensenellales_CAG-74, and *Oscillospiraceae* showed high relative abundances within the high methanogen phenotype subjects regardless of their age (Fig. 3C, Supplementary Fig. 6A, B, Supplementary Material 1).

The microbial composition of those with a high methanogen phenotype was particularly similar in YAs and OAs. In contrast, CENT exhibited slightly varied microbial composition for the high methanogen phenotype, highlighting a distinct microbial ecosystem within this age group. Particularly, a noteworthy observation was made within CENT, where *Gemmiger* and *Faecalibacterium* as well as *Ruminococcaceae* demonstrated a marked reduction in subjects with the high methanogen phenotype (Fig. 3C and D, Supplementary Fig. 6A, B, Supplementary Material 1). On the other hand, *Ruminococcaceae* showed increased relative abundance in subjects with high methanogen phenotype in YAs and OAs. Interestingly, although not statistically significant, *Ruminococcus_E* was highly abundant in subjects with high methanogen phenotype within all three age groups (Fig. 3D, Supplementary Fig. 6B, Supplementary Material 1), which was consistent with previous reports [17]. *Ruminococcus* demonstrates a significant correlation with dietary fibers, owing to its efficient breakdown of microcrystalline cellulose. Previous studies conducted by researchers have indeed elucidated a link between cellulose degradation and the subsequent emission of methane [56].

### Co-occurring bacterial consortium of predominant *Methanobrevibacter* spp. Is complex and dynamic in subjects with high methanogen phenotype across all age groups

When examining the microbial networks of *M. smithii* and *Ca.* M. intestini in individuals exhibiting a high methanogen phenotype, it became apparent that the stability of the microbial networks for these two archaeal species remained consistent in these subjects, irrespective of age categorization. Each network displayed comparable complexity. Interestingly, the network complexity and co-occurring microbes were mostly similar in subjects with high methanogen phenotype within YAs and CENT. The microbiome composition and co-occurring taxa are often linked to health status. In aging, OAs commonly experience inflammaging, a chronic low-grade inflammation where the microbiome plays a pivotal role [57]. Centenarians may exhibit a unique microbial profile associated with superior health and longevity, reflecting a more similar composition of co-occurring taxa.

The betweenness and closeness centrality metrics for *M. smithii* and *Ca.* M. intestini exhibited comparable patterns to those seen in all subjects, irrespective of the presence of high methanogen phenotype (Table 1). Among YAs with high methanogen phenotype, *Ca.* M. intestini played a central role in the microbial network, whereas in OAs and CENT with high methanogen phenotype, *M. smithii* emerged as the primary driver of the microbial network (Table 1).

Taking a closer look at *M. smithii* and *Ca.* M. intestini edges within networks, mutual exclusion of these archaeal species with members of *Ruminococcaceae*, *Bacteroidaceae*, and *Streptococcaceae*/*Streptococcus*, and co-presence with Oscillospirales/*Oscillospiraceae* as well as Christensenellales were observed (Supplementary Fig. 7), which was similar to the trends observed before, irrespective of the presence or absence of high methanogen phenotype.

Of note, while most members of *Lachnospiraceae*, including *Acetatifactor* (YAs, CENT), *Agathobacter* (all age groups), *Blautia* (all age groups), *Dorea* (all age groups), *Eubacterium* (YAs, CENT), *Fusicatenibacter* (all age groups), *Lachnospira* (YAs, CENT), and *Roseburia* (YAs, CENT), showed co-presence with *M. smithii* and *Ca.* M. intestini, some of the genera within this family including *Eisenbergiella* (all age groups) and *Mediterranibacter* (all age groups), showed mutual exclusion with these archaeal species. This could be attributed to potential different ecological roles or metabolic functions of these bacteria leading to competition or niche differentiation as well as difference in their adaptation to specific environmental conditions.

### High methanogen phenotype is associated with the upregulation of genes involved in butyrate and propionate production

The decrease in *Lachnospiraceae*, known butyrate producers, has been consistently documented in CENT in several studies, irrespective of the geographical region [13, 58, 59]. This prompts the hypothesis that individuals having detectable *M. smithii* and *Ca.* M. intestini in their gut microbiome may better cope with decline during aging, as evidenced by the consistent co-occurrence of these archaeal species with *Oscillospiraceae*, another known butyrate-producing component of the gut microbiota. Notably, an increased relative abundance of *Oscillospiraceae* was observed in subjects exhibiting a high methanogen phenotype (Fig. 2, Supplementary Figs. 6, 7).

**Fig. 4:**
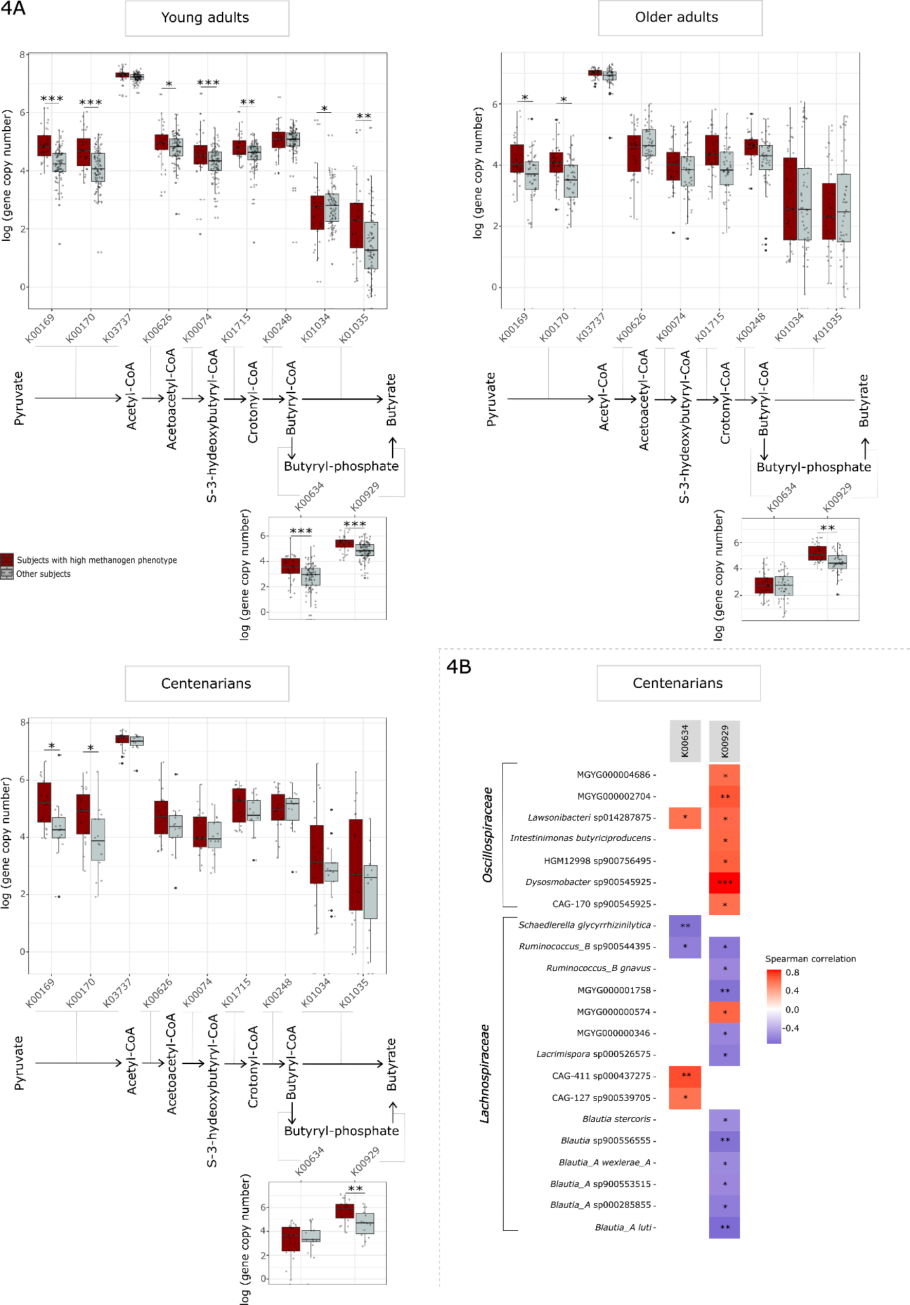
(**A**) Boxplot of gene copy numbers (metagenomic abundance) involved in butyrate production. K00169, K0017, K03737: Pyruvate ferredoxin oxidoreductase [EC: 1.2.7.1]; K00626: Acetyl-CoA acetyltransferase [EC: 2.3.1.9]; K00074: 3-hydroxylbutyryl-CoA dehydrogenase [EC: 1.1.1.157]; K01715: Enoyl-CoA hydratase [EC: 4.2.1.17]; K00248: butyryl-CoA dehydrogenase [EC: 1.3.8.1]; K01034, K01035: Acetate-CoA transferase [EC: 2.8.3.8]; K00634: phosphate butyryltransferase [EC: 2.3.1.19]; K00929: butyrate kinase [EC: 2.7.2.7]. Significance levels are indicated as ***q < 0.001, **q < 0.01, *q < 0.05, for differentially abundance testing by DESeq2. (**B**) Correlation of butyrate kinase pathway genes in centenarians with bacterial taxa. ***q < 0.001, **q < 0.01, *q < 0.05

Diverse and abundant genes related to butyrate metabolism are present in the metagenomic datasets. Specifically, pathways such as the butyryl-CoA: acetate-CoA pathway and the butyrate kinase pathway contribute to the formation of butyrate. Genes encoding enzymes directly involved in butyrate production were analyzed and individuals with a high methanogen phenotype exhibited varying levels of these genes (Fig. 4A).

In YAs, the majority of genes involved in both pathways, were significantly elevated in individuals exhibiting a high methanogen phenotype compared to other subjects (Fig. 4A). In OAs and CENT, only the genes associated with pyruvate ferredoxin oxidoreductase (K00169, K00170) were significantly elevated (q < 0.05) within the butyryl-CoA: acetate-CoA pathway, though most other genes in this pathway also showed an increased level (Supplementary Material 1). Intriguingly, in subjects with high methanogen phenotype within these age groups, butyrate kinase (K00929), the terminal enzyme in the butyrate kinase pathway responsible for synthesizing butyrate, exhibited statistically elevated levels (q < 0.01) and the gene coding for phosphate butyryltransferase (K00634) displayed an increasing trend (though not reaching statistically significance), hinting at the potential significance of the butyrate kinase pathway in the elevated butyrate levels in these individuals (Fig. 4A).

Both butyrate kinase and acetate-CoA transferase are key enzymes in butyrate production, and butyrate kinase levels were significantly higher in all age groups in individuals with a high methanogen phenotype (Supplementary Material 1). A closer examination revealed a positive correlation between butyrate production genes via the butyrate kinase pathway and *Oscillospiraceae*, while a negative correlation was observed with *Lachnospiraceae* (Fig. 4B, Supplementary Fig. 8). This observation supports our hypothesis that individuals with a high methanogen phenotype, may compensate for the reduction in butyrate levels by harboring elevated levels of *Oscillospiraceae*, particularly through the butyrate kinase pathway.

## Discussion

Aging-associated changes in the gut microbiome have been tackled in several studies [60], however, despite the vital importance of the gut archaeome, there is a significant gap in our understanding regarding how age, and especially longevity, affects the distribution of archaea within the gut and vice versa. Specifically, the role of methanogenic archaea with a profound impact on the structure and functionality of the entire gastrointestinal microbiome remains poorly elucidated. Moreover, since *Methanobrevibacter smithii*, as the predominant archaeal species within the human gut, has been recently divided into two clades, namely *M. smithii* and *Candidatus* Methanobrevibacter intestini, highly resolved investigation of gut archaeome helps to deepen our understanding of the association of these two archaeal species with aging and their role in the emergence of high methanogen phenotype.

Our study revealed that, with aging, the gut archaeome richness and/or diversity decreased, which was in contrast to the previous reports based on 16S rRNA gene sequencing [15]. We then delved more into the differences of the archaeal profile of different age groups. The increased relative abundance of *M. smithii* with advancement of age, and the possible increased relative abundance of this methanogen prior to longevity has been previously reported [6].

Similarly, our study suggests a trend where the relative abundance of Methanobacteria generally shows increased abundance in older age groups. However, we observed that OAs in our study showed a higher relative abundance of *Ca*. M. intestini compared to YAs and CENT, rather than *M. smithii*. It is important to note that these observations are based on cross-sectional data. Hence, observed differences could be also influenced by varying health statuses among the studied age groups.

Thermoplasmata is another commonly found archaeal taxon in the human gut that tends to be more abundant in OAs. This has led to the hypothesis that since these archaea have environmental origins, age could play a role in promoting their survival in certain individuals [16]. Our observations also indicated the high relative abundance of these archaea in OAs compared with YAs, however, interestingly, the relative abundance of Thermoplasmata in CENT showed reduction as compared with OAs and was observed to be rather comparable to YAs. There is evidence suggesting that Thermoplasmata could potentially contain genes responsible for producing certain metabolites such as methylglyoxal, indole, and acetaldehyde with the potential of disrupting DNA or signaling pathways [61, 62]. Although further molecular experiments and longitudinal studies are required to fortify the involvement of these archaea in disease progression, there might be a potential link between the presence of Thermoplasmata and disease, which could explain why they appear to be less abundant in individuals with a longer lifespan. Interestingly, according to the literature, within this archaeal order, certain species may counteract trimethylamine (TMA, involved in the progression of atherosclerosis), while some species lack this capability. On the other hand, the high relative abundance of *Ca*. Methanomassilicocales intestinalis in frail individuals raises questions about its role and it is not clear whether its high relative abundance is favored due to factors like altered gut transit or independently contributing to inflammation. Further research is needed to assess the immune potential of dominant Methanomassiliicoccales, especially those using TMA, and determine if their role is primarily positive through TMA removal or more nuanced [48].

According to the archaeal abundance, subjects could be stratified into low and high methanogen phenotypes. Our study revealed that the prevalence of subjects with high methanogen phenotype increases with age. Interestingly, not only *M. smithii*, but also *Ca.* M. intestini was found to contribute to the emergence of high methanogen phenotype, with a more evident contribution of *Ca.* M. intestini in OAs, which can be argued by the increased relative abundance of this archaeal species in this age group. The exact cause of the link between the higher prevalence of high methanogen phenotype in older age remains somewhat elusive, however, the slower digestive transit times often observed in aging individuals, as well as their differences in dietary habits and contact with livestock could contribute to the overrepresentation of archaea [63, 64]. Moreover, the centenarians lifelong adherence to traditional dietary patterns, such as the Mediterranean diet [65] likely plays a significant role in shaping their gut microbiome and the overrepresentation of methanogens like *Methanobrevibacter* and the subsequent higher prevalence of high methanogen phenotypes. One key factor is the high intake of dietary fiber from plant-based foods, which supports the breakdown of complex carbohydrates into C1 metabolites. These metabolites serve as substrates for *Methanobrevibacter*, fueling methanogenesis and resulting in higher methane emissions in the gut [17]. However, a notable limitation of our study is the lack of precise nutritional data for each sample. This constraint prevented a more detailed analysis of the specific dietary contributions to high methanogen prevalence in each age group and gut archaeome composition. Additionally, the association between *Methanobrevibacter* and a lean phenotype [66, 67] may also help explain its higher abundance in centenarians, since Italian centenarians remained physically active throughout their lives, maintaining lean body types [65].

Subjects with high methanogen phenotype showed different microbiome signatures and higher microbial diversity, especially evident in YAs and OAs rather than CENT. Interestingly, a significantly higher alpha diversity has been frequently linked to improved stability and resistance to disruptions [68]. This increased diversity of microbial species in subjects with high methanogen phenotype has been previously linked to the ability of methanogens to reduce the hydrogen partial pressure and thus facilitating the microbial fermentation, which is otherwise restricted by the hydrogen accumulation and inhibition of NAD coenzyme regeneration [69]. In CENT with high methanogen phenotype, only the richness index was significantly higher and not the evenness. In general, despite occasional contradictions [70], lower gut microbial alpha diversity has been shown in CENT compared to that of YAs and OAs [12, 71]. A variety of confounding factors can influence the controversial reports regarding microbiota alpha diversity with respect to aging. These factors include host and/or lifestyle factors as well as geography or the number of included subjects in cohorts. Moreover, although the CENT cohort represents a healthy population, the process of aging, particularly in its later stages, is associated with a natural decline in gastrointestinal function and the host immune response. This decline may contribute to the onset of chronic low-grade inflammation and metabolic disorders [72–74]. Therefore, a reduction in alpha diversity measures of high methanogen phenotype in CENT compared to those within YAs and OAs is not surprising.

When examining the relationship between the bacterial taxa associated with *M. smithii* or *Ca.* M. intestini in different age groups, we observed that in CENT, these species had more complex networks with other bacterial taxa compared to YAs and OAs, which was more akin to those seen in individuals with a high methanogen phenotype, who showed a dynamic archaea-bacteria network. Upon closer examination of these networks, we found that *M. smithii* or *Ca.* M. intestini consistently co-occurred regardless of the age group, which was in contrast to previous findings based on Sanger sequencing [75]. Additionally, the family *Christensenellaceae* was consistently associated with these archaeal species across all age groups. The co-occurrence of *Christensenella* with *M. smithii* or *Ca.* M. intestini was consistent with previous research that demonstrated a mutually beneficial relationship between *Christensenella* and *Methanobrevibacter* through interspecies hydrogen transfer [76]. *Methanobrevibacter* spp. play a crucial role in efficiently digesting complex polysaccharides by optimizing hydrogen levels for bacterial polysaccharide digestion and consuming the end products of bacterial fermentation. In our analysis of bacteria-archaea networks, we identified *Oscillospiraceae* known for butyrate production [77, 78] and subsequent anti-inflammatory properties to co-occur with both *M. smithii* and *Ca.* M. intestini. This suggests a mutualistic or syntrophic relationship between these gut bacteria and archaea. Conversely, we observed a significant negative association between these archaeal species and members of the *Lachnospiraceae* family, which are also known butyrate producers in the gut [79]. The mutual exclusion between *Lachnospiraceae* and methanogens is not surprising, given that *Lachnospiraceae* functions as acetogens, utilizing hydrogen and carbon dioxide in the gut to produce acetate. In contrast, although some methanogens, such as *Methanosarcina*, can use acetate as the sole energy source, the predominant gut methanogens employ hydrogen and carbon dioxide for methanogenesis. Consequently, these microorganisms engage in a competitive relationship for substrates [80, 81].

Interestingly, the cumulative presence of *Lachnospiraceae*, recognized as butyrate-producing bacteria, decreases with age [13]. Our findings reveal that individuals with a high methanogen phenotype in the OAs and CENT age group exhibit elevated levels of genes (metagenome) associated with the butyrate production pathway, particularly the butyrate kinase pathway. Our correlation analysis highlights a positive association between the gene responsible for butyrate kinase, a pivotal enzyme in the butyrate production pathway, and members of *Oscillospiraceae*. This underscores the significance of these microbial taxa in butyrate production, a known health-promoting factor, among individuals with a high methanogen phenotype. Hence, it can be inferred that these individuals may compensate for the decline in *Lachnospiraceae* not only through the co-occurrence of *M. smithii* and Ca. *M. intestini* with *Oscillospiraceae* but also through an increased abundance of the latter in their microbiota. Consequently, it is plausible to suggest that, as individuals age, the reduction in *Lachnospiraceae* (potentially due to dietary factors) could be more manageable for those with high methanogen phenotype, as indicated by comparable butyrate levels to the microbiome of subjects lacking this phenotype. Therefore, having high methanogen phenotype could be potentially associated with the maintenance of optimal butyrate levels in the gut.

Another interesting observation was the consistent negative co-occurrence of *M. smithii* or *Ca.* M. intestini with *Streptoccocus*, especially in OAs. It is noteworthy to mention that increased levels of metabolic makers of dysregulation has been associated with increased abundance of *Streptococcus* and therefore it is mostly linked with unhealthy aging [82], suggesting that the presence of these methanogens might be associated with healthy aging rather than the progression of disease. However, more longitudinal studies are required to confirm this hypothesis.

## Conclusion

This study again supports the relevance of the archaeal microbiome component on human physiology and aging. Our research highlights the dynamic age-related associations in methanogen composition, and particularly the higher prevalence of the high methanogen phenotype in centenarians. Our study emphasizes the significance of *Ca.* M. intestini, evident in its surge in older adults, its co-occurrence with *M. smithii*, and its substantial role in subjects with high methanogen phenotype. This is the first insight into the critical role of this new archaeal representative for the human host. Moreover, our findings underscore the importance of methanogens partnering with specific butyrate-producing bacteria using the butyrate kinase pathway, enhancing the health status of individuals with high methanogen phenotype.

## Electronic supplementary material

Below is the link to the electronic supplementary material.

Supplementary Material 1

Supplementary Material 2: Fig. 1. Conceptual outline of study cohorts. Three different study populations were used. For Cohort A, stool samples collected for a study by Kumpitsch et al. [17] were used. Cohorts A and B were collected from the same location but at different time points and from different subjects. Samples from Cohorts A and B were processed the same way and a similar method for library preparation was employed. Subjects within Cohort C were enrolled in a study with a close location to cohorts A and B by Rampelli et al. [19], and the deposited sequences were used for further evaluations. In order to mitigate the study effect and remove the bias based on the methods employed in sequencing, ConQuR was used for correcting the read counts. Fig. 2. Principal Coordinate Analysis (PCoA) plots were generated to visualize the clustering of study cohorts based on Bray-Curtis and Aitchison dissimilarity computed using raw count data. Each data point on the plot corresponds to a sample, while each ellipse represents a batch (study cohorts A, B, or C), with the centroid denoting the mean. The size of the ellipse reflects the dispersion of data points within each batch, and the angle of the ellipse indicates higher-order characteristics specific to the batch. Furthermore, the ellipse connects the 95th percentile of data points, providing a visual representation of the batch’s overall distribution. Fig. 3. Alpha and beta diversity indices of overall microbiome in different age groups (A) Evenness and richness indices tended to decrease with aging. T-test was used for statistical analysis of the richness index due to the normal distribution of the values while evenness values were not normally distributed. (B) NMDS analysis shows a shift of the clusters based on aging. Statistical significance is indicated by **p < 0.001**p < 0.01 and *p < 0.05. C). Fig. 4. Age-dependent prevalence of high methanogen phenotype. (A) The prevalence of high methanogen phenotype increases with age. (B) Association plot visualizing that high frequency of high methanogen phenotype is associated with the CENT age group rather than other age groups. Area of the box is proportional to the difference in observed and expected frequencies of the presence of high methanogen phenotype. The baseline (dotted line) indicates independence of high methanogen phenotype to aging. The boxes rising above the baseline indicate that the observed frequency of a cell is greater than the expected one (if the data were random), and vice versa. Cells representing negative residuals are drawn below the baseline and vice versa. The width of each of the bar elements in the mosaic reflects the relative magnitude of its value. Fig. 5. An examination of the richness index revealed significant differences based on the presence of high methanogen phenotype irrespective of the age classification, with those with high methanogen phenotype showing significantly higher richness. However, the evenness index was only significantly higher in the presence of high methanogen phenotype in YAs. ***p < 0.001, **p < 0.01. Fig. 6. Box plot of CLR-transformed abundances of the top bacterial taxa per age group based on the presence of high methanogen phenotype. (A) Top bacterial taxa at family level per age group. (B) Top bacterial taxa at genus level. Significance levels are indicated as ***q < 0.001, **q < 0.01, *q< 0.05, for differentially abundance testing by ALDEx. Fig. 7. SparCC co-occurrence networks of *M. smithii* and *Ca.* M. intestini in samples with high methanogen phenotype in different age groups of YAs, OAs, and CENT (2 A). Positive and negative SparCC co-occurrences are indicated in green and black, respectively. The details of these co-occurrences are shown in more detail in (2B). Fig. 8. Correlation of butyrate kinase pathway genes in young adults with bacterial taxa. ***q < 0.001, **q < 0.01, *q < 0.05.

## Data Availability

Raw nucleotide data generated and used in the study (Cohorts A and B) can be found in the Sequence Read Archive under the project accession PRJEB72212. All scripts, bracken output, and all the relevant metadata for the cohorts are provided in (https://github.com/Roxy-mzh/Archaea_And_Aging).

## Abbreviations

*M. smithii*: *Methanobrevibacter smithii*
*Ca*. M. intestini: *Candidatus* Methanobrevibacter instestini
YAs: Young adults
OAs: Older adults
CENT: Centenarians
