# Supplementary material for: Age-related dynamics of predominant methanogenic archaea in the human gut microbiome
Source: BMC Microbiol. 2025 Apr 4;25:193. doi: 10.1186/s12866-025-03921-9 (PMC11969853; doi:10.1186/s12866-025-03921-9)
Supplement: Supplementary file 2 — Supplementary Material 2: Fig. 1. Conceptual outline of study cohorts. Three different study populations were used. For Cohort A, stool samples collected for a study by Kumpitsch et al. [17] were used. Cohorts A and B were collected from the same location but at different time points and from different subjects. Samples from Cohorts A and B were processed the same way and a similar method for library preparation was employed. Subjects within Cohort C were enrolled in a study with a close location to cohorts A and B by Rampelli et al. [19], and the deposited sequences were used for further evaluations. In order to mitigate the study effect and remove the bias based on the methods employed in sequencing, ConQuR was used for correcting the read counts. Fig. 2. Principal Coordinate Analysis (PCoA) plots were generated to visualize the clustering of study cohorts based on Bray-Curtis and Aitchison dissimilarity computed using raw count data. Each data point on the plot corresponds to a sample, while each ellipse represents a batch (study cohorts A, B, or C), with the centroid denoting the mean. The size of the ellipse reflects the dispersion of data points within each batch, and the angle of the ellipse indicates higher-order characteristics specific to the batch. Furthermore, the ellipse connects the 95th percentile of data points, providing a visual representation of the batch’s overall distribution. Fig. 3. Alpha and beta diversity indices of overall microbiome in different age groups (A) Evenness and richness indices tended to decrease with aging. T-test was used for statistical analysis of the richness index due to the normal distribution of the values while evenness values were not normally distributed. (B) NMDS analysis shows a shift of the clusters based on aging. Statistical significance is indicated by **p < 0.001**p < 0.01 and *p < 0.05. C). Fig. 4. Age-dependent prevalence of high methanogen phenotype. (A) The prevalence of high methanogen phenotype increases [file 12866_2025_3921_MOESM2_ESM.docx]

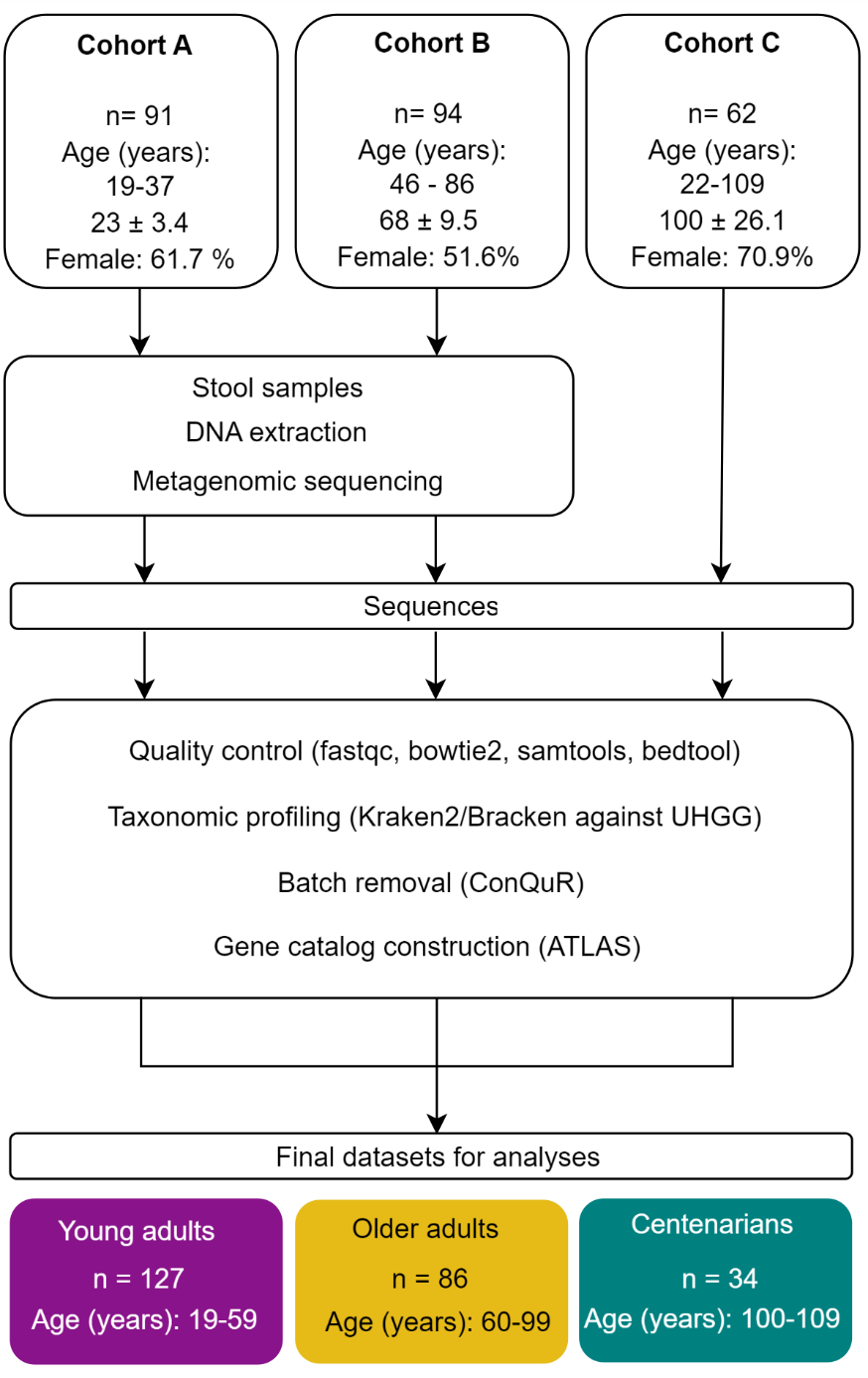


Supplementary Fig. 1. Conceptual outline of study cohorts. Three different study populations were used. For Cohort A, stool samples collected for a study by Kumpitsch et al. (17) were used. Cohorts A and B were collected from the same location but at different time points and from different subjects. Samples from Cohorts A and B were processed the same way and a similar method for library preparation was employed. Subjects within Cohort C were enrolled in a study with a close location to cohorts A and B by Rampelli et al. (19), and the deposited sequences were used for further evaluations. In order to mitigate the study effect and remove the bias based on the methods employed in sequencing, ConQuR was used for correcting the read counts.


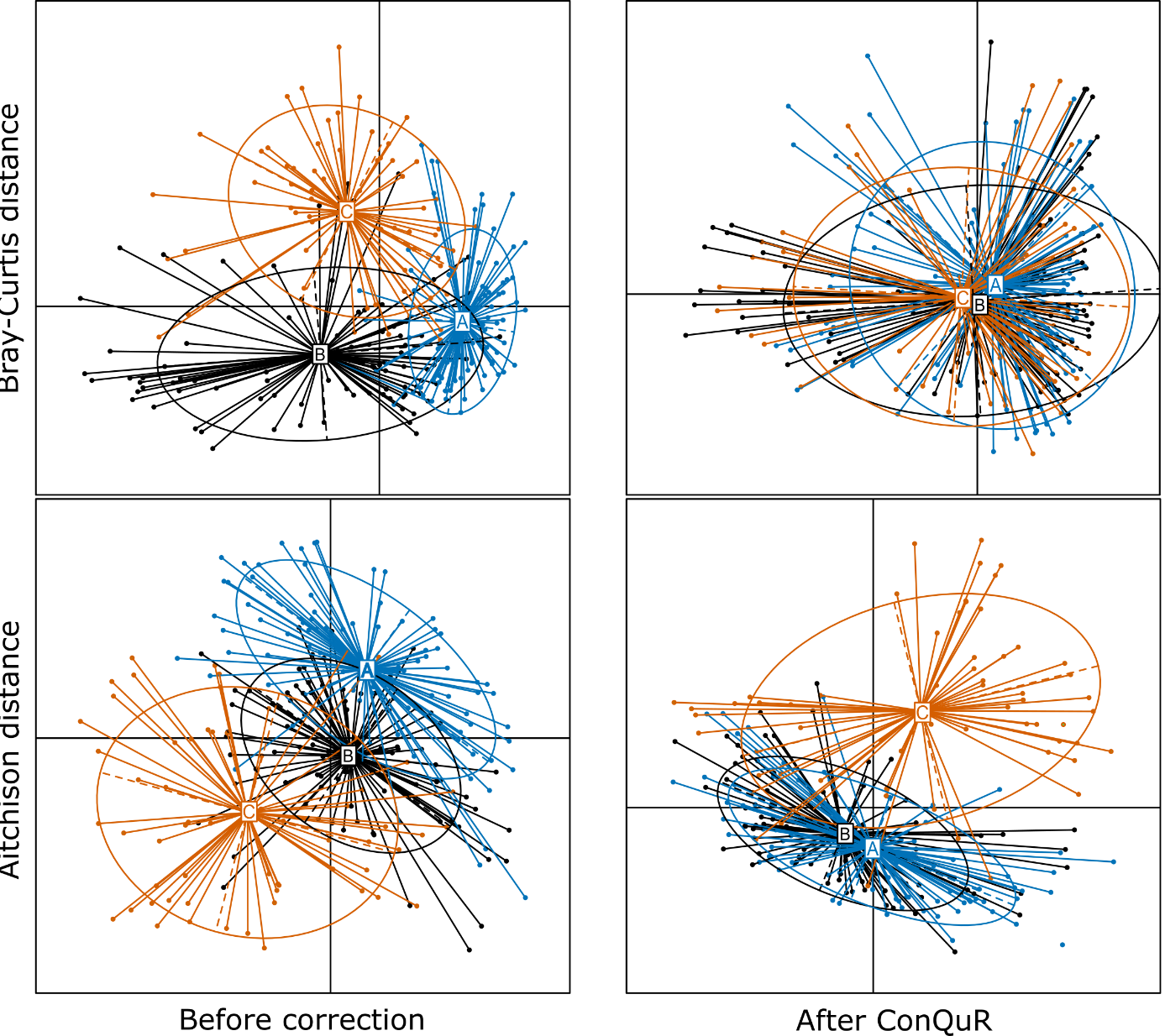


Supplementary Fig. 2.  Principal Coordinate Analysis (PCoA) plots were generated to visualize the clustering of study cohorts based on Bray-Curtis and Aitchison dissimilarity computed using raw count data. Each data point on the plot corresponds to a sample, while each ellipse represents a batch (study cohorts A, B, or C), with the centroid denoting the mean. The size of the ellipse reflects the dispersion of data points within each batch, and the angle of the ellipse indicates higher-order characteristics specific to the batch. Furthermore, the ellipse connects the 95th percentile of data points, providing a visual representation of the batch's overall distribution.


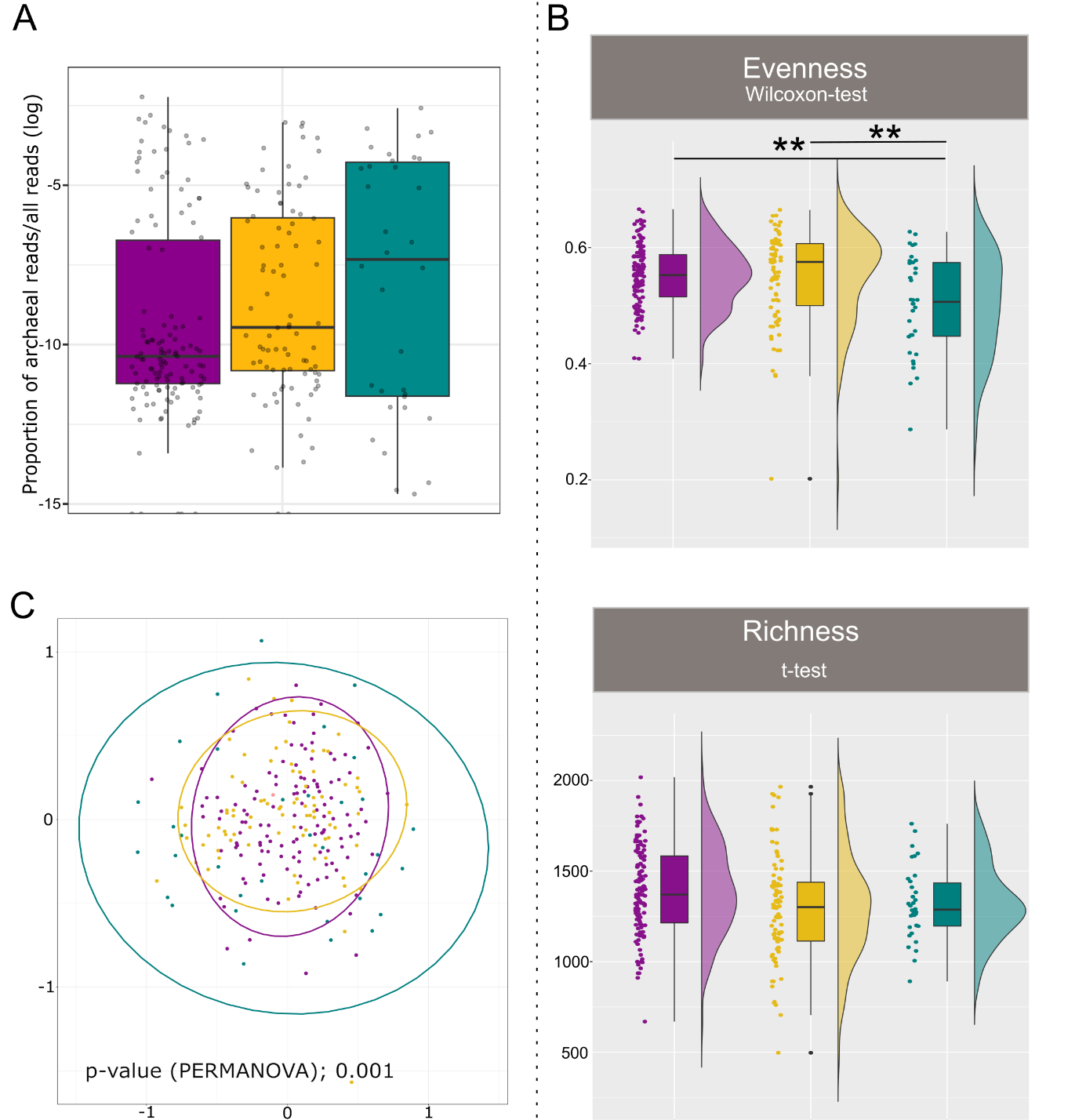


Supplementary Fig .3. Alpha and beta diversity indices of overall microbiome in different age groups A) Evenness and richness indices tended to decrease with aging. T-test was used for statistical analysis of the richness index due to the normal distribution of the values while evenness values were not normally distributed. B) NMDS analysis shows a shift of the clusters based on aging. Statistical significance is indicated by ***p* < 0.001***p* < 0.01 and **p* < 0.05. C).


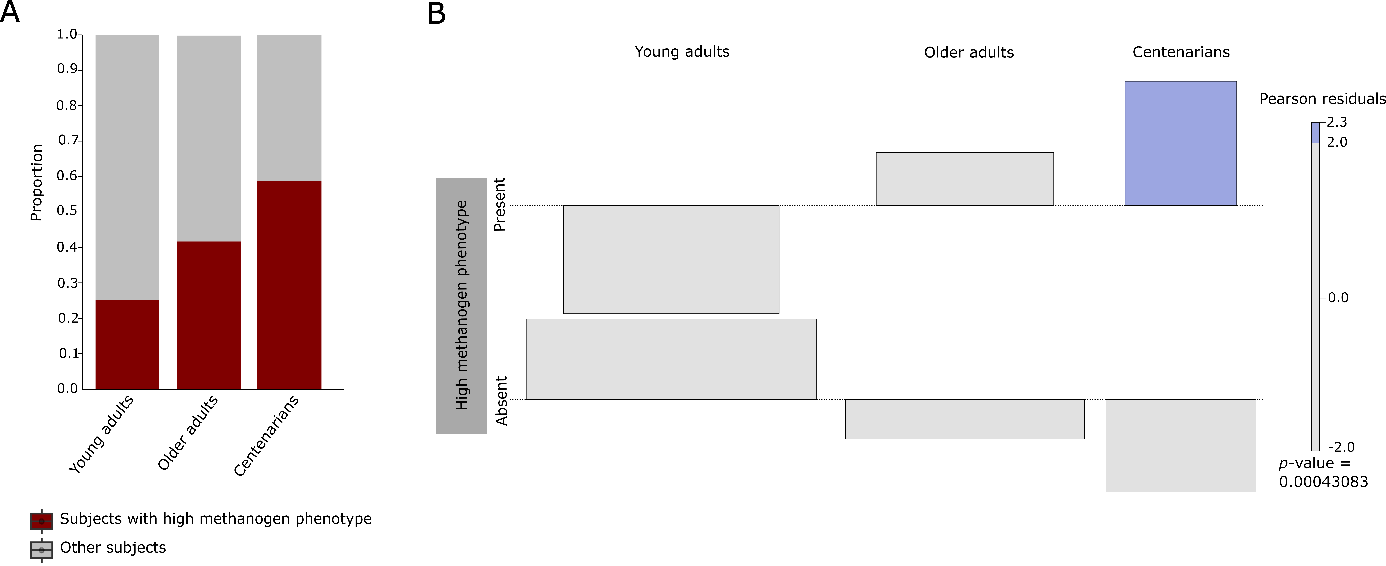


Supplementary Fig. 4. Age-dependent prevalence of high methanogen phenotype. A) The prevalence of high methanogen phenotype increases with age. B) Association plot visualizing that high frequency of high methanogen phenotype is associated with the CENT age group rather than other age groups. Area of the box is proportional to the difference in observed and expected frequencies of the presence of high methanogen phenotype. The baseline (dotted line) indicates independence of high methanogen phenotype to aging. The boxes rising above the baseline indicate that the observed frequency of a cell is greater than the expected one (if the data were random), and *vice versa*. Cells representing negative residuals are drawn below the baseline and vice versa. The width of each of the bar elements in the mosaic reflects the relative magnitude of its value.


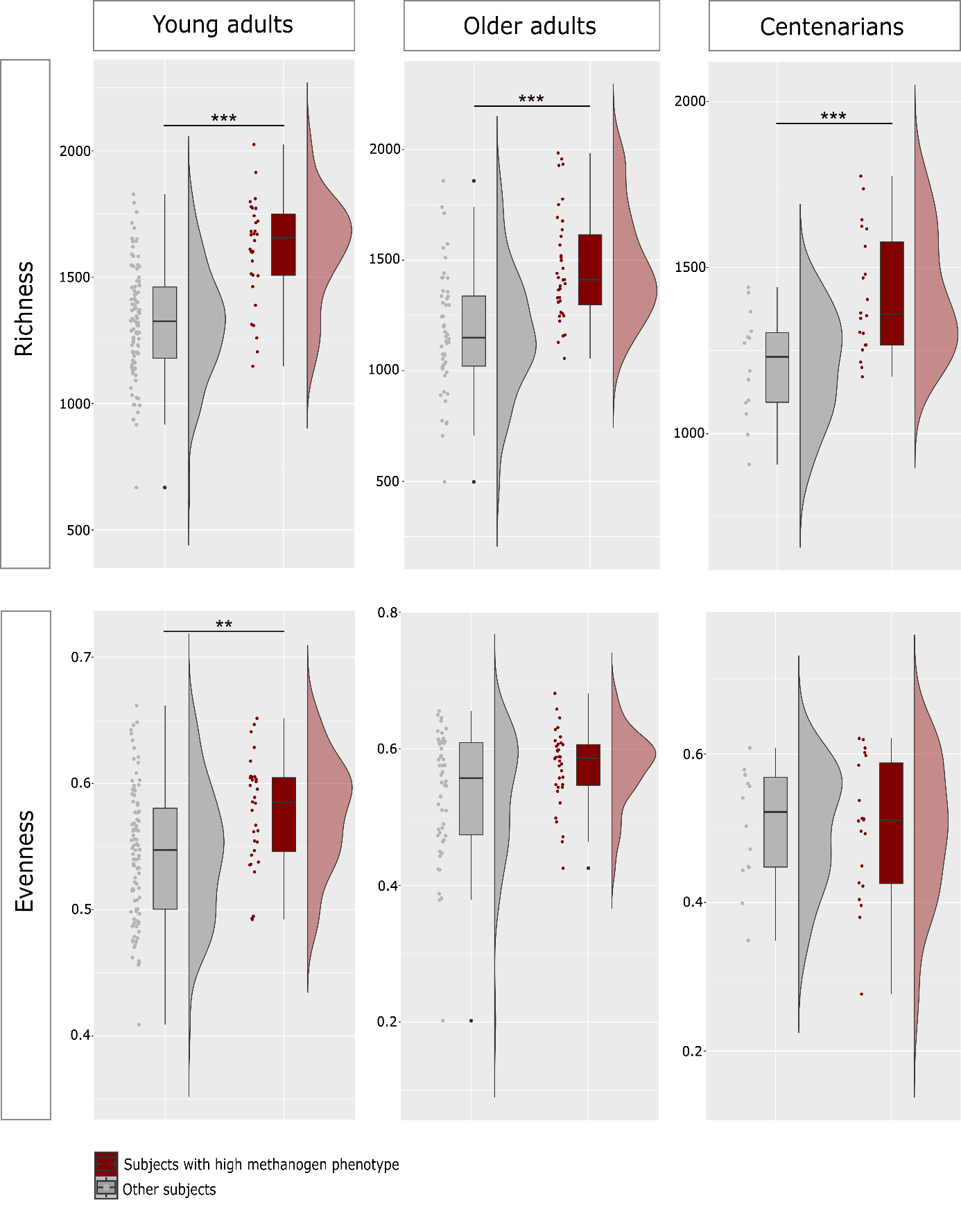


Supplementary Fig. 5. An examination of the richness index revealed significant differences based on the presence of high methanogen phenotype irrespective of the age classification, with those with high methanogen phenotype showing significantly higher richness. However, the evenness index was only significantly higher in the presence of high methanogen phenotype in YAs. ***p< 0.001, **p< 0.01.


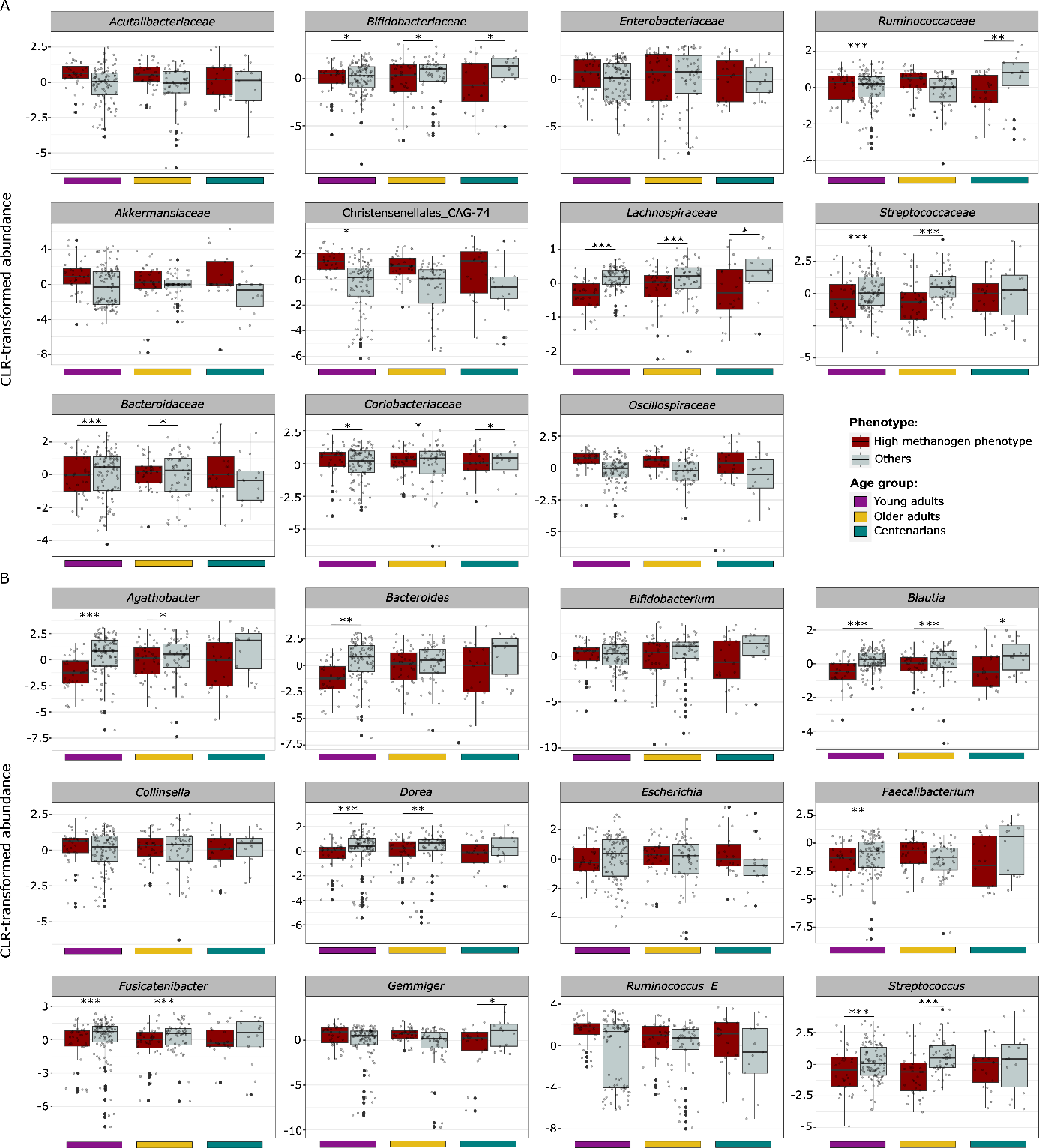
Supplementary Fig. 6. Box plot of CLR-transformed abundances of the top bacterial taxa per age group based on the presence of high methanogen phenotype. A) Top bacterial taxa at family level per age group. B) Top bacterial taxa at genus level. Significance levels are indicated as ***q < 0.001, **q < 0.01, *q< 0.05, for differentially abundance testing by ALDEx.


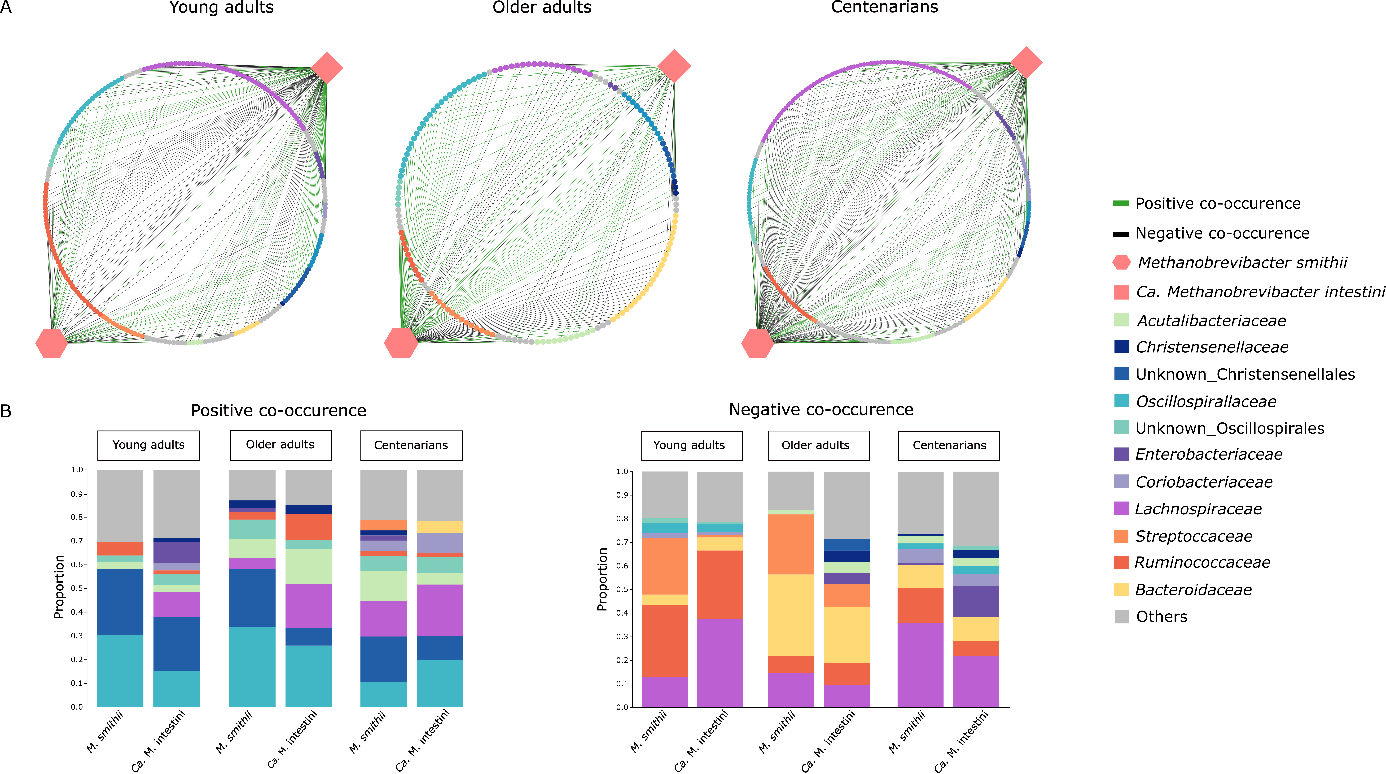
Supplementary Fig. 7. SparCC co-occurrence networks of *M. smithii* and *Ca.* M. intestini in samples with high methanogen phenotype in different age groups of YAs, OAs, and CENT (2A). Positive and negative SparCC co-occurrences are indicated in green and black, respectively. The details of these co-occurrences are shown in more detail in (2B).


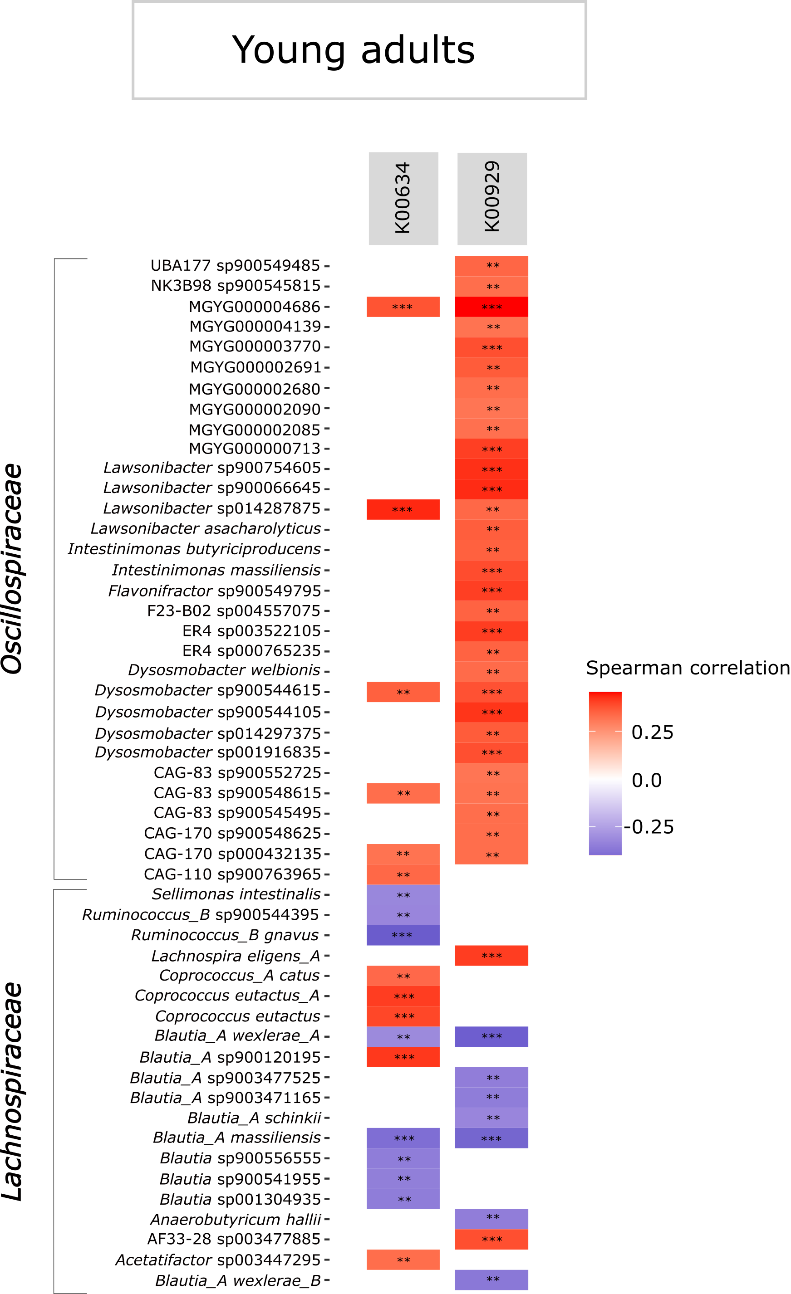


Supplementary Fig. 8. Correlation of butyrate kinase pathway genes in young adults with bacterial taxa. ***q < 0.001, **q < 0.01, *q< 0.05.
